# Ensemble Machine Learning on Bulk RNA-Seq Identifies 17-Gene Signature Predicting Neoadjuvant Chemotherapy Response in Breast Cancer

**DOI:** 10.3390/cimb48010094

**Published:** 2026-01-16

**Authors:** Stelios Lamprou, Styliana Georgiou, Triantafyllos Stylianopoulos, Chrysovalantis Voutouri

**Affiliations:** 1 AnaBioSi-Data Ltd., Athalassas Ave 176—MyOffice 204, Strovolos 2025, Cyprus; stelios@anabiosi-data.com (S.L.); styliana@anabiosi-data.com (S.G.); 2 Cancer Biophysics Lab., Department of Mechanical Engineering, University of Cyprus, P.O. Box 20537, Nicosia 1678, Cyprus; tstylian@ucy.ac.cy

**Keywords:** breast cancer, biomarkers, digital pathology, precision oncology, precision medicine

## Abstract

Predicting neoadjuvant chemotherapy response in breast cancer remains critical for optimizing treatment strategies, yet robust predictive biomarkers are lacking. This study implemented an ensemble machine learning approach to identify a gene expression signature predicting pathological complete response (pCR) versus residual disease (RD) using bulk RNA-sequencing data from GSE163882 (138 RD, 80 pCR). We employed TMM normalization with differential expression analysis (250 genes, FDR < 0.05, |log2FC| ≥ 1), ensemble feature selection across five classifiers (Random Forest, Gradient Boosting, SVM, k-NN, and Neural Network) with 10-fold repeated cross-validation, and stacked ensemble development. Consensus selection identified a 17-gene signature consistently ranked across algorithms. The stacked ensemble achieved 0.97 AUC post-testing on hold-out test data. External validation on the independent GSE240671 cohort (37 pCR, 25 RD) following ComBat batch correction achieved ROC AUC of 0.78 and PR AUC of 0.85 with isotonic calibration, demonstrating balanced accuracy of 0.71 and 0.86 sensitivity for pCR detection. Pathway enrichment revealed associations with cell cycle regulation (E2F3, MKI67), DNA repair (BRCA2), and transcriptional control (MED1), with six priority genes (MED1, BRCA2, E2F3, PITPNB, H1-1, and FARP2) showing established breast cancer relevance. This externally validated 17-gene signature provides a biologically grounded tool for NAC response prediction in precision oncology.

## 1. Introduction

Breast cancer remains the most commonly diagnosed cancer and a leading cause of cancer-related mortality among women worldwide [1]. Surgery has traditionally been the cornerstone of breast cancer management, yet it carries risks, including anesthesia-related complications, surgical morbidity, potential for increased metastatic dissemination due to intraoperative tumor cell shedding, and postoperative immune suppression [2,3].

Neoadjuvant chemotherapy (NAC) is a systemic therapy administered before surgical intervention and it has emerged as an attractive alternative or adjunct to surgery, offering several clinical advantages [4]. NAC can downstage tumors, enabling breast-conserving surgery instead of mastectomy and reducing the extent of axillary lymph node dissection, thereby improving cosmetic outcomes and quality of life [5]. Importantly, clinical trials have demonstrated equivalent overall survival (OS) and disease-free survival (DFS) between neoadjuvant and adjuvant chemotherapy, with NAC offering the added benefit of in vivo assessment of tumor chemosensitivity [6].

Achieving pathological complete response (pCR), which is defined as the absence of invasive carcinogenic tissue in the breast and lymph nodes following NAC, strongly correlates with improved long-term survival outcomes, particularly in aggressive subtypes such as triple-negative and HER2-positive breast cancer [7]. Patients achieving pCR demonstrate significantly reduced hazard ratios for DFS and OS, with some studies reporting hazard ratios as low as 0.34 for OS in pCR patients compared to those with residual disease [8]. Furthermore, emerging evidence suggests that patients with pCR may have favorable outcomes even with de-escalated or omitted surgery in select cases, as systemic therapy may eliminate micro-metastatic disease without the potential tumor-disseminating effects of surgical manipulation [9]. However, a significant proportion of patients do not respond adequately to NAC, experiencing unnecessary toxicity, treatment delays, and progression to inoperable disease [10].

Current methods to predict NAC response rely on clinical parameters (tumor size, grade, and receptor status) and radiological assessments, which demonstrate limited sensitivity and specificity for individualized treatment stratification [11]. Molecular biomarkers and multi-gene expression signatures have shown promise in prognostication but remain limited by reproducibility issues, high costs, and lack of validation across diverse populations. Bulk RNA sequencing captures comprehensive transcriptomic profiles and provides rich, high-dimensional data suitable for discovering predictive signatures; however, translating such data into robust, clinically actionable models remains a significant challenge [12].

Machine learning (ML) techniques offer powerful computational frameworks to analyze complex genomic data and identify robust biomarkers for treatment response prediction [13]. Nevertheless, many studies rely on single classification algorithms, which are susceptible to model instability, overfitting, and limited generalizability across independent cohorts [14]. Ensemble learning strategies that combine predictions from multiple algorithms can enhance predictive accuracy, stability, and external validity [15].

In this study, we developed a robust pipeline architecture by employing rigorous cross-validation, as well different data splitting rationales, and selecting genes for the final signature that were obtained from ensemble feature selection. We then developed and externally validated an ensemble ML pipeline integrating five classification algorithms that fall in four different categories: decision tree-based ensemble algorithms (Random Forest (RF) and Gradient Boosting (GB)), kernel methods (Support Vector Machines (SVMs)), instance-based learning algorithms (k-Nearest Neighbors (KNN)), and deep learning (DL) algorithms (1-layer Neural Networks (NNs)), to identify a stable, biologically interpretable 17-gene signature predictive of pCR versus residual disease (RD) in breast cancer patients undergoing NAC. We leveraged publicly available bulk RNA-seq datasets (GSE163882 for discovery and GSE240671 for external validation), which provided sufficient sample sizes and outcome annotations for robust model training and testing.

Breast cancer was selected as the primary application due to its high prevalence, well-established NAC protocols, and availability of high-quality transcriptomic datasets with treatment response annotations. However, we hypothesize that the ensemble ML framework developed here is universally applicable to other solid tumors treated with neoadjuvant regimens, including lung, colorectal, and gastric cancers, where predictive biomarkers for treatment response remain critically needed. By demonstrating strong discrimination (Area Under the Curve (AUC) 0.78) and calibration on an independent validation cohort, our 17-gene signature addresses critical gaps in NAC response prediction and provides a scalable, biologically grounded tool for precision oncology.

## 2. Materials and Methods

### 2.1. Study Design and Data Sources

This retrospective multi-cohort study was designed to develop and externally validate a gene expression-based classifier of NAC response in breast cancer. The pipeline comprised a discovery phase, leveraging bulk RNA-seq data from the publicly available GEO dataset GSE163882 [16], and an external validation phase using GSE240671 [17]. The discovery cohort included 218 breast cancer patients treated with NAC, classified into pathological complete response (pCR, *n* = 80) and residual disease (RD, *n* = 138). The validation cohort included 62 breast cancer patients, classified into pCR (*n* = 37) and RD (*n* = 25) using Residual Cancer Burden categories and clinical notes. Phenotype and clinical metadata were retrieved using the GEOquery R package (v2.78.0) [18]. Only cases with definitive response phenotypes (pCR or extreme RD) were included in the validation phase.

#### Sample Size Justification

Sample sizes were determined by the availability of publicly accessible datasets meeting strict inclusion criteria: documented NAC treatment with confirmed pCR or RD outcomes, high-quality bulk RNA-sequencing data, and appropriate clinical annotations. The discovery cohort (GSE163882) and validation cohort (GSE240671) represent all available samples in these datasets fulfilling these requirements. For the final 17-gene signature, the events-per-variable (EPV) ratio was 4.7 in the discovery phase. The events-per-variable (EPV) ratio was calculated as follows:

(1) $$EPV=\frac{n_{pCR}}{p}$$

where n_pCR_ is the number of patients achieving pathological complete response and p is the number of genes in the predictive signature.

For the discovery phase, the EPV = 80/17 = 4.7.

While the EPV ratio for the 17-gene signature is below traditional thresholds (EPV ≥ 10) [19], recent research indicates that EPV criteria poorly predict model performance when modern validation methods are used [20]; specifically, we employed rigorous cross-validation (repeated 10-fold across four data splits), bootstrap resampling (*n* = 2000 iterations), and ensemble feature selection to prevent overfitting.

### 2.2. Data Preprocessing and Normalization

Raw RNA-seq count data were processed in R (v4.2+) using the edgeR package (v4.8.0). Library sizes were normalized via trimmed mean of M-values (TMM) to minimize sample-specific biases while preserving biological variability [21,22]. Gene counts were transformed to log2 counts-per-million (CPM) with a prior count of 1. Quality assessment, filtering, and sample-level expression matrix construction were performed with edgeR [23] and custom scripts. Batch effect correction across discovery and validation cohorts was performed using the ComBat algorithm in the sva package (v3.58.0) [24], harmonizing gene expression distributions between datasets.

### 2.3. Differential Expression and Feature Selection

Differential expression analysis compared pCR and RD samples using the DESeq2 package (v1.50.0) [25], with false discovery rate (FDR) correction. Genes meeting significance criteria (FDR < 0.05, |log2FC| ≥ 1) were retained for model development. Ensemble feature selection employed five ML algorithms: RF (ranger (v0.17.0) [26]), GB (gbm (v2.2.2) [27]), SVM (svmLinear), KNN (knn), and NN (nnet). Models were implemented via the caret package (v7.0-1) [28] with four train/test splits (60/40, 70/30, 80/20, and 90/10) (i.e., 20 models in total) and 10× repeated 10-fold cross-validation (100 Random Repeats). Prior to any data allocation for model development, testing, and validation, the same data allocation was used with the aid of the set.seed() function to ensure robust comparability between outputs. Up-sampling was applied using the Rose package (v0.0-4) [29] to balance classes during training, preventing the models from being biased towards the majority class (i.e., RD) and improving their performance on the minority class (i.e., pCR). Feature importance was aggregated across algorithms; genes ranked as top predictors in the best-performing model from each ML method (i.e., five models in total) formed the consensus signature. The best-performing model was identified by comparing performance metrics and the AUC (calculated using the pROC package (v1.19.0.1) [30]) by detecting significant differences (i.e., *p*-value < 0.05) using DeLong’s test [31] or by qualitative assessment where differences in metrics were not significant.

### 2.4. Classifier Development and Evaluation

Consensus genes were used to train a Stacked Elastic Net ensemble classifier (meta-learner approach; glmnet (v 4.1-10) [32]) and averaging ensembles. Prior to the development of the stacked classifier, five base classifiers (i.e., RF, GB, SVM, KNN, and NN) were trained with parallel processing, using the doParallel package (v1.0.17) [33], on the entire dataset to obtain the best-tune for each and extract out-of-fold predictions that were used as the meta-features to train the stacked classifier. The stacked classifier, as well as the base models, was then trained on 80% of the data and 10-fold cross-validation. Hold-out set performance was assessed with accuracy, sensitivity, specificity, and positive and negative predictive values (PPV/NPV). Robustness was quantified via bootstrap resampling (*n* = 2000), producing 95% confidence intervals for all metrics. Ensemble models were evaluated against the base classifiers using area under the receiver operating characteristic curve (ROC AUC) and DeLong’s test.

### 2.5. External Validation Phase

The trained classifier was evaluated in the independent validation cohort (GSE240671), after normalization, batch correction, and harmonization. Performance metrics included ROC AUC, precision–recall AUC, balanced accuracy, sensitivity, and specificity. The validation phase was performed using isotonic regression, to obtain calibrated probabilities that accurately reflect the true event likelihood, with calibration curves visualized using verification (v.1.45) [34] and ggplot2 (v4.0.1) [35].

### 2.6. Bioinformatics and Pathway Analysis

Consensus signature genes were investigated for biological relevance via pathway enrichment analysis, querying Gene Ontology (GO), KEGG, and Reactome databases through the Enrichr web tool (https://maayanlab.cloud/Enrichr/, accessed on 2 October 2025) [36]. The literature review further characterized priority genes and associated molecular functions.

### 2.7. Data and Code Availability

All datasets are publicly available through NCBI GEO: GSE163882 (discovery) and GSE240671 (validation). Analysis scripts, pipelines, and gene lists are available in the repository at https://github.com/stelioslamprou37/cancer-nac-ensemble (accessed on 20 November 2025).

## 3. Results

### 3.1. Cohort Characteristics and Differential Expression Analysis

**Figure 1:**
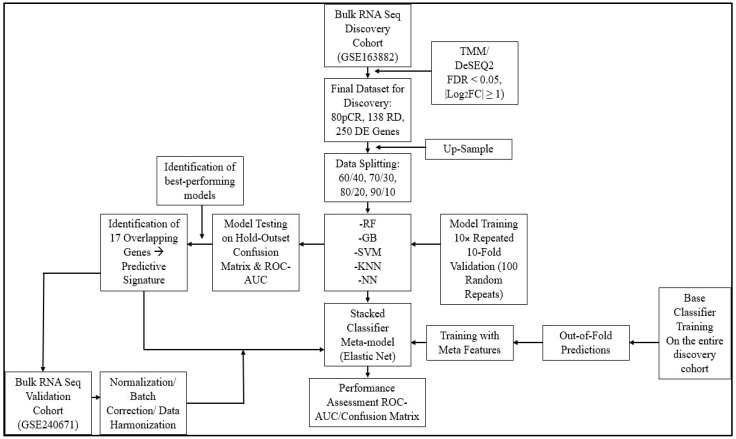
Machine learning workflow for identification of predictive gene signature from RNA-seq data. The study utilized a bulk RNA-seq discovery cohort (GSE163882) comprising 80 complete responders (pCR), 138 RD decliners, and 250 differentially expressed (DE) genes identified through TMM/DeSEQ2 normalization (FDR < 0.05, |Log2FC| ≥ 1). Data splitting strategies (60/40, 70/30, 80/20, and 90/10) were implemented with up-sampling to address class imbalance. Four machine learning algorithms (RF: Random Forest, GB: Gradient Boosting, SVM: Support Vector Machine, and NN: Neural Network) were evaluated through 10× repeated 10-fold cross-validation (100 random repeats). Base classifiers were trained on the entire discovery cohort, generating out-of-fold predictions used as meta-features for the stacked classifier meta-model (Elastic Net). Model performance was assessed using ROC-AUC and confusion matrix metrics on the hold-out test set. The workflow identified 17 overlapping genes constituting the final predictive signature, which was subsequently validated on an independent bulk RNA-seq validation cohort (GSE240671) following normalization and batch correction procedures.

The study design and workflow are presented in Figure 1. The discovery cohort (GSE163882) comprised 218 breast cancer patients treated with NAC, of whom 80 (36.7%) achieved pCR and 138 (63.3%) exhibited residual disease (RD). Following TMM normalization and quality filtering, differential expression analysis using DESeq2-identified genes was significantly differentially expressed between pCR and RD groups (FDR < 0.05, |log2 FC| ≥ 1). These genes served as input features for ensemble ML analysis. The external validation cohort (GSE240671) consisted of 62 breast cancer patients stratified by Residual Cancer Burden into pCR (*n* = 37) and extreme RD (*n* = 25) categories. Patients with ambiguous or moderate RD were excluded to establish a stringent binary outcome classification.

### 3.2. Multi-Algorithm Model Training and Performance Comparison

Five supervised learning algorithms (i.e., RF, GB, SVM, KNN, and NN) were trained independently on the 250 differentially expressed genes using four training/testing configurations (60/40, 70/30, 80/20, and 90/10 splits). Each model underwent 10-times repeated 10-fold cross-validation to optimize hyperparameters and assess generalization capacity. Performance evaluation across all training/testing splits identified three configurations demonstrating superior and consistent performance across all five classifiers: Model Configuration 1 (80/20 split), Model Configuration 2 (70/30 split), and Model Configuration 4 (90/10 split). Model Configuration 3 (60/40 split) demonstrated inferior performance across multiple classifiers and was excluded from subsequent analysis. Performance metrics for all models including the top five models and their configurations are presented in Table 1. Based on a performance metrics qualitative assessment and significance assessment using DeLong’s test, the best-performing models were as follows:

- RF 1 (80/20 split);
- GB 4 (90/10 split);
- SVM 2 (70/30 split);
- KNN 2 (70/30 split);
- NN 4 (90/10 split).

**Table 1:** Performance metrics across machine learning models and data splitting strategies.

Metric		Random	Forest			Gradient	Boosting			SVM				Neural	Network			KNN		
	60/40	70/30	80/20	90/10	60/40	70/30	80/20	90/10	60/40	70/30	80/20	90/10	60/40	70/30	80/20	90/10	60/40	70/30	80/20	90/10
Accuracy	0.833	0.793	0.692	0.824	0.815	0.805	0.72	0.853	0.796	0.77	0.729	0.765	0.815	0.793	0.72	0.912	0.685	0.736	0.636	0.618
Balanced Accuracy	0.834	0.793	0.694	0.835	0.816	0.805	0.722	0.854	0.797	0.771	0.73	0.761	0.816	0.793	0.722	0.921	0.696	0.734	0.644	0.644
Sensitivity	0.821	0.814	0.6	0.737	0.786	0.814	0.636	0.842	0.786	0.884	0.691	0.79	0.786	0.814	0.655	0.842	0.393	0.581	0.346	0.421
Specificity	0.846	0.773	0.789	0.933	0.846	0.796	0.808	0.867	0.808	0.659	0.769	0.733	0.846	0.773	0.789	1	1	0.886	0.942	0.867
NPV	0.815	0.81	0.651	0.737	0.786	0.814	0.677	0.813	0.778	0.853	0.702	0.733	0.786	0.81	0.683	0.833	0.605	0.684	0.577	0.542
PPV	0.852	0.778	0.75	0.933	0.846	0.796	0.778	0.889	0.815	0.717	0.76	0.79	0.846	0.778	0.768	1	1	0.833	0.864	0.8
AUC	0.93	0.88	0.83	0.91	0.9	0.87	0.86	0.96	0.81	0.87	0.79	0.87	0.83	0.84	0.798	0.93	0.74	0.77	0.73	0.76

NPV = Negative Predictive Value; PPV = Positive Predictive Value; AUC = Area Under the Receiver Operating Characteristic Curve.

#### Feature Importance Analysis and Top Gene Extraction

**Figure 2:**
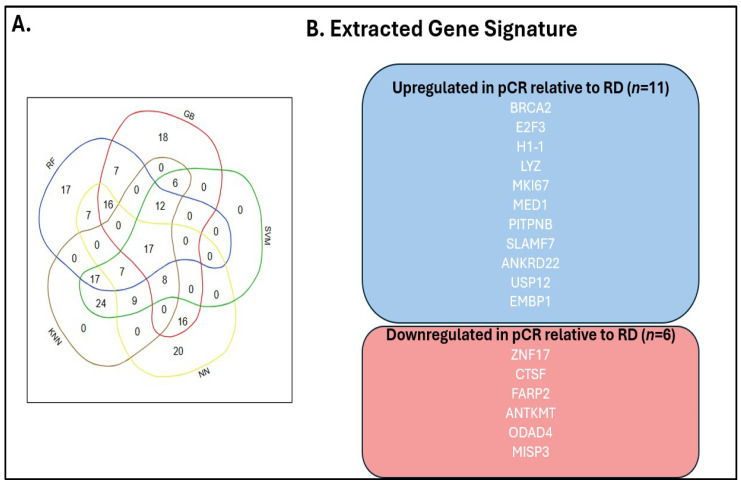
Identification of a 17-gene predictive signature through ensemble machine learning analysis. The Venn diagram (**A**) illustrates the overlap of gene features selected by five machine learning algorithms: Random Forest (RF), Gradient Boosting (GB), Support Vector Machine (SVM), Neural Network (NN), and K-Nearest Neighbors (KNN). Numbers within each section represent the count of algorithm-specific genes, with 17 genes appearing across all five models (center intersection). (**B**) displays the composition of the extracted 17-gene signature, stratified by expression patterns in pCR versus RD samples. Eleven genes (BRCA2, E2F3, H1-1, LYZ, MKI67, MED1, PITPNB, SLAMF7, ANKRD22, USP12, and EMBP1) showed upregulation (blue) in pCR relative to RD samples, while six genes (ZNF17, CTSF, FARP2, ANTKMT, ODAD4, and MISP3) demonstrated downregulation (red) in pCR samples. These genes constitute the final predictive signature used for downstream validation analyses.

Feature importance scores were extracted from each trained classifier across the five best-performing models. For each configuration, the top 100 genes ranked by aggregated importance scores across all five algorithms were identified. While individual algorithms demonstrated some variation in feature rankings, substantial overlap was observed in the top-ranked genes across classifiers, indicating convergence on a core set of biologically relevant predictors. To identify a robust, stable gene signature independent of a training/testing split and classification algorithm, we performed strict consensus filtering: only genes appearing in the top 100 ranked features of all five classifiers were retained. This stringent cross-algorithm, cross-configuration consensus approach identified 17 genes consistently selected as top predictors. In total, 17 genes were found to be overlapped in all five models, as shown in Figure 2. The 17-gene consensus signature comprised the following:

Upregulated in pCR relative to RD (*n* = 11): BRCA2, E2F3, H1-1, LYZ, MKI67, MED1, PITPNB, SLAMF7, ANKRD22, USP12, and EMBP1.

Downregulated in pCR relative to RD (*n* = 6): ZNF17, CTSF, FARP2, ANTKMT, ODAD4, and MISP3.

### 3.3. Discovery Phase Classifier Performance

A stacked ensemble classifier was constructed using the 17-gene consensus signature, integrating predictions from the five base classifiers through a meta-learning framework trained on Model Configuration 1 (80/20 split). An averaging ensemble approach was also evaluated for comparison. Both ensemble strategies were evaluated on an independent hold-out test set (*n* = 54; 27 pCR, 27 RD) that was not used during training or feature selection. The stacked ensemble classifier confusion matrix revealed the following:

- True pCR predictions: 22;
- True RD predictions: 25;
- False RD predictions (Type I error): 2;
- False pCR predictions (Type II error): 5.

Bootstrap resampling (*n* = 2000 iterations) quantified uncertainty in performance metrics:

- Accuracy: 0.90 (SD = 0.046, 95% CI: 0.78–0.94);
- Sensitivity: 0.81 (SD = 0.076, 95% CI: 0.70–0.96);
- Specificity: 0.93 (SD = 0.051, 95% CI: 0.80–1.00);
- NPV: 0.80 (SD = 0.067, 95% CI: 0.70–0.96);
- PPV: 0.95 (SD = 0.058, 95% CI: 0.77–1.00).

**Figure 3:**
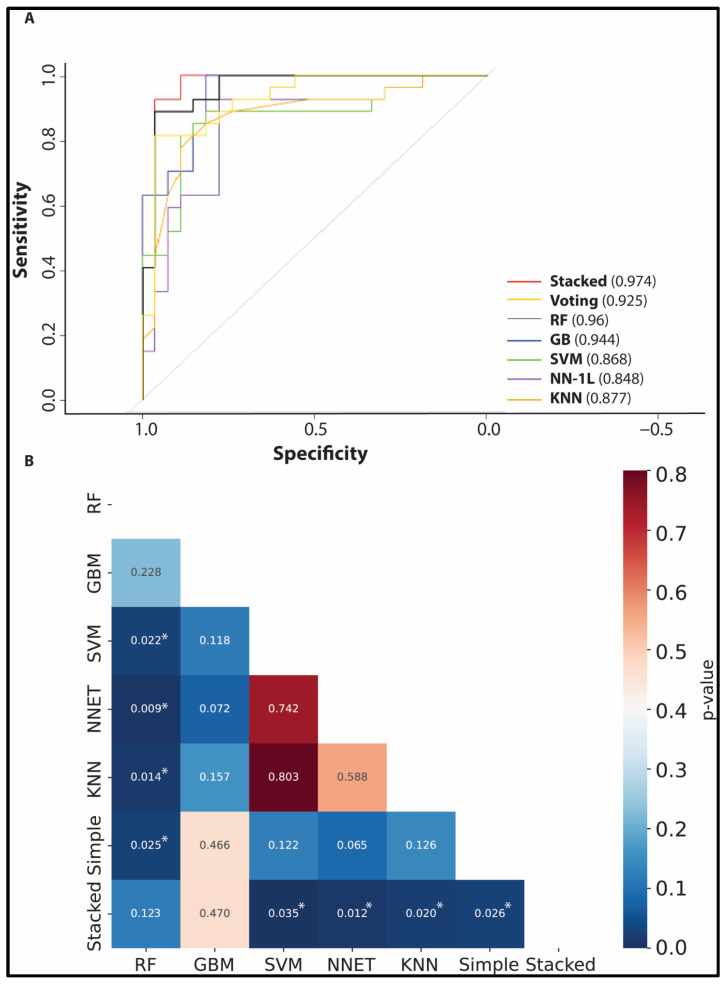
Discovery phase performance: ROC curves comparing the stacked ensemble (AUC = 0.97) with individual base classifiers and an averaging ensemble (**A**), with DeLong test *p*-values (i.e., significant: *p*-value < 0.05—denoted with *) annotated (**B**) (Voting and Simple are the same classifier).

Narrower confidence intervals for accuracy and specificity indicated stable overall performance, whereas wider sensitivity intervals reflected variability in true positive detection, likely attributable to limited sample size in the pCR class. The stacked ensemble achieved an area under the receiver operating characteristic curve (ROC AUC) of 0.97 on the hold-out test set. DeLong’s test confirmed (see Figure 3) statistically significant superior performance of the stacked ensemble compared to all individual base classifiers (*p* < 0.05 for all pairwise comparisons), apart from RF and GB. The voting-simple ensemble showed comparable but slightly inferior performance (AUC = 0.94), supporting the selection of the stacked approach for external validation.

### 3.4. External Validation Cohort Performance

The trained stacked ensemble classifier was applied to the independent validation cohort (GSE240671, *n* = 62) to assess generalizability beyond the discovery dataset. All 17 genes obtained from the discovery cohort were measured in the validation cohort. Differential expression analysis revealed that all 17 genes followed the same direction of expression as in the discovery cohort analysis (i.e., same gene was upregulated or downregulated in pCR relative to RD for the two cohorts). However, differential expression was only significant for two genes: LYZ (Log2FC = 2.21, FDR = 0.002) and MED1 (Log2FC = 1.28, FDR = 0.049).

Given the technical heterogeneity between discovery and validation cohorts (different sequencing platforms, library preparation protocols, and clinical centers), batch effect correction was performed using the ComBat algorithm to harmonize expression distributions while preserving biological variation. Also, the same normalization process was carried out as in the discovery set.

**Figure 4:**
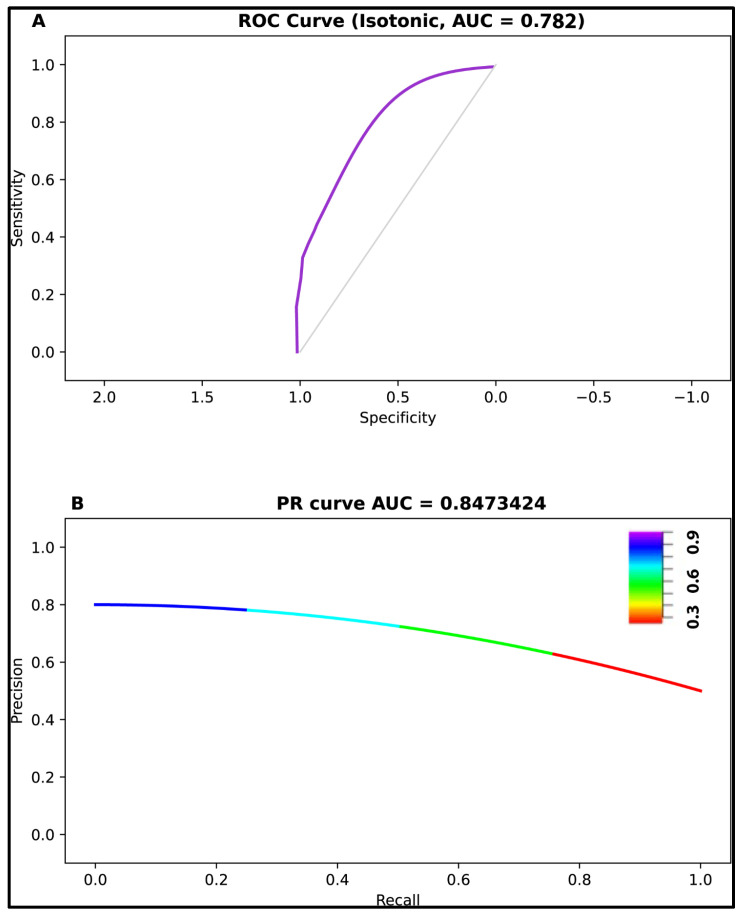
External validation performance: (**A**) ROC curve (AUC = 0.78); (**B**) precision–recall curve (AUC = 0.85).

Following batch correction, predicted class probabilities from the stacked ensemble were calibrated with isotonic regression calibration, producing well-calibrated probabilities with an ROC-AUC of 0.78 (uncalibrated ROC-AUC: 0.74) and precision–recall curve (AUC = 0.85), as shown in Figure 4. External validation bootstrapped (*n* = 2000) performance metrics were also obtained:

- Accuracy: 0.74 (95% CI: 0.63–0.83, SD: 0.05);
- Balanced Accuracy: 0.71 (95% CI: 0.6–0.8, SD: 0.06);
- Sensitivity (pCR detection): 0.86 (95% CI: 0.74–0.95, SD: 0.06);
- Specificity (RD detection): 0.56 (95% CI: 0.4–0.7, SD: 0.1).

### 3.5. Pathway Enrichment Analysis

To elucidate the biological mechanisms underlying the 17-gene signature’s predictive capacity, comprehensive pathway enrichment analysis was performed using Enrichr, querying four major biological databases: Gene Ontology Biological Process (GO BP) 2023, Gene Ontology Molecular Function (GO MF) 2023, KEGG 2021 Human Pathways, and Reactome 2022 Pathways.

Of the 17 genes in the consensus signature, six genes (E2F3, BRCA2, MED1, PITPNB, H1-1, and FARP2) exhibited significant pathway enrichment (adjusted *p* < 0.15) across multiple databases. The remaining 11 genes showed limited enrichment in the queried databases, suggesting either novel functional roles or participation in biological processes not yet comprehensively annotated in current pathway resources. Notably, E2F3 demonstrated the most extensive enrichment profile (eight pathways), followed by BRCA2 (seven pathways) and MED1 (five pathways) as shown in Table 2. Table 3 presents the top significant biological processes and pathways identified.

Specifically, E2F3 was enriched in eight pathways spanning transcription initiation, cell cycle regulation (G2 phase, G1/S-related processes, and fat cell proliferation), apoptosis modulation, and cancer pathways, including pancreatic, breast, and bladder cancer, as well as cellular senescence, underscoring its central role in proliferation-related chemotherapy sensitivity. BRCA2 mapped to seven pathways primarily linked to DNA repair and genome maintenance, including homologous recombination, Fanconi anemia, and HDR via alternative end-joining, together with telomere localization and cancer pathways such as breast and pancreatic cancer, highlighting its involvement in double-strand break repair and treatment response. MED1 was enriched in five pathways related to transcriptional regulation and nuclear receptor signaling, including transcription initiation at RNA polymerase II promoters and binding to ligand-activated nuclear receptors (LBD, PPAR, and retinoic acid receptors), supporting a role in hormone-signaling crosstalk during NAC response. PITPNB contributed via two molecular function terms associated with phosphatidylinositol and phosphatidylcholine transfer activities, suggesting links between lipid transport, membrane dynamics, drug handling, and stress responses. H1-1 (histone H1.1) was associated with cellular senescence and apoptosis-induced DNA fragmentation, consistent with chromatin remodeling events during chemotherapy-induced cell death. FARP2 showed enrichment in a single Reactome pathway, SEMA3A–plexin repulsion signaling via inhibition of integrin adhesion, implicating cytoskeletal and adhesion remodeling as additional components of the NAC response mechanism captured by the signature.

## 4. Discussion

This study developed and externally validated a 17-gene signature for predicting NAC response in breast cancer using an ensemble ML approach integrating five independent classification algorithms. The stacked ensemble classifier achieved strong discrimination in the discovery cohort (AUC = 0.97) and demonstrated robust generalization to an independent validation cohort (ROC AUC = 0.78, PR AUC = 0.85, balanced accuracy = 0.71). Pathway enrichment analysis revealed biologically coherent mechanisms involving DNA repair, cell cycle regulation, and transcriptional control, with six priority genes demonstrating established roles in breast cancer biology.

### 4.1. Comparison with Existing NAC Response Prediction Studies

Previous studies have developed gene expression signatures for NAC response prediction in breast cancer, but many suffer from limitations, including reliance on single classification algorithms, lack of external validation, or use of proprietary platforms limiting clinical translation [37]. Moreover, a comparison of our 17-gene signature with the shortlisted genes reported in the original study [16] revealed limited direct overlap. Only LYZ and CTSF, both markers of innate immune activity, were shared between the two analyses. The published study’s signature was predominantly enriched for immune-related genes involved in T-cell activation, cytokine signaling, and chemokine-mediated immune recruitment, whereas our gene set was mainly composed of tumor-intrinsic markers associated with cell cycle regulation, DNA repair, transcriptional control, and metabolic processes. These findings suggest that while both approaches capture biologically relevant determinants of treatment response, they emphasize distinct but potentially complementary mechanisms.

To that extent, recent ML approaches have shown promise for NAC response prediction. A 12-gene signature developed using LASSO regression and validated in independent cohorts demonstrated good discriminative capacity [38]. Another study identified a 10-gene signature associated with recurrence risk and chemotherapy response in ER-positive breast cancer [39]. None of these genes were found to overlap with our 17-gene signature. However, these studies typically employ single-feature selection methods and limited external validation cohorts, raising concerns about reproducibility and generalizability.

Our approach addresses these limitations through several methodological advances. First, ensemble feature selection across five algorithms (RF, GB, SVM, k-NN, and NN) and multiple data configurations ensure identification of stable, reproducible features rather than algorithm-specific artifacts. Recent work has demonstrated that ML biomarker discovery suffers from severe reproducibility issues, with an average of 93% of features failing to replicate across independent datasets when single-method selection is employed [40]. Ensemble methods that require consensus across multiple algorithms and data partitions substantially improve reproducibility [41]. Second, external validation in an independent cohort processed at a different institution with different sequencing protocols provides strong evidence for generalizability. Third, the use of publicly available data and open-source algorithms enhance reproducibility and enable rapid clinical implementation without proprietary barriers.

#### Comparison with Established Assays

The 17-gene signature developed in this study differs fundamentally in purpose and clinical context from established multigene assays, such as Oncotype DX and MammaPrint, which were designed and validated to predict recurrence risk and guide adjuvant treatment decisions in hormone receptor-positive, HER2-negative breast cancer [37,42]. Both Oncotype DX and MammaPrint have subsequently demonstrated associations with pathological complete response in the neoadjuvant setting, with high-risk scores predicting increased the likelihood of pCR [42]. However, these assays were not optimized for neoadjuvant response prediction [42]. In contrast, our 17-gene signature was specifically developed to distinguish complete responders from non-responders across all breast cancer molecular subtypes, not restricted to hormone receptor-positive cases, reflecting the broader application of NAC in contemporary clinical practice [37]. The gene composition differs markedly, with our signature enriched for proliferation and replication stress markers, such as MKI67, E2F3, and BRCA2, whereas Oncotype DX emphasizes estrogen receptor signaling and proliferation genes, and MammaPrint focuses on cell cycle and metastasis-related pathways [37].

While established assays provide valuable information that can inform prognostic decisions, our signature aims to address the distinct clinical question of identifying patients most likely to achieve complete tumor eradication with neoadjuvant chemotherapy, potentially guiding treatment intensification or de-escalation strategies in pre-operative settings.

### 4.2. Biological Interpretation and Mechanistic Insights

The 17-gene signature demonstrates biological coherence, with six genes exhibiting significant pathway enrichment in cancer-relevant processes. The upregulation of BRCA2 in pCR samples initially appears counterintuitive, as germline BRCA2 mutations and BRCA2 deficiency are well-established predictors of increased chemotherapy sensitivity due to impaired homologous recombination DNA repair [43]. However, our findings of elevated BRCA2 expression in chemotherapy-responsive tumors align with emerging evidence that BRCA2 expression levels, distinct from loss-of-function mutations, reflect complex biology. Studies have shown that tumors with BRCA2 mutations demonstrate enhanced sensitivity to DNA-damaging chemotherapy through failure to repair double-strand breaks, with overall response rates exceeding those of sporadic breast cancers treated with anthracycline-based regimens [43]. However, the relationship between BRCA2 expression levels and chemotherapy response may reflect proliferative capacity and replication stress, where tumors with higher BRCA2 expression exhibit enhanced DNA repair that paradoxically increases sensitivity to replication-interfering agents through improved resolution of replication–transcription conflicts [44]. Alternatively, high BRCA2 expression in pCR samples may indicate functional DNA repair capacity that enables effective processing of chemotherapy-induced lesions through error-free pathways, preventing mutagenesis-driven resistance.

The observed association between elevated BRCA2 expression and pathological complete response requires careful biological interpretation. This finding appears paradoxical given that germline BRCA2 mutations and loss-of-function confer chemotherapy sensitivity through impaired homologous recombination repair. However, our observation of high BRCA2 expression likely reflects proliferative capacity and constitutive replication stress rather than DNA repair proficiency. BRCA2 plays an essential role in suppressing endogenous replication stress during normal DNA replication, and its expression is tightly linked to cell cycle progression and S-phase entry [45]. The co-occurrence of elevated BRCA2 with MKI67 and E2F3 in our signature strongly suggests that these tumors exhibit high proliferative indices with accompanying replication stress [46]. Highly proliferative tumors with constitutive replication stress and dependence on DNA replication machinery are intrinsically vulnerable to chemotherapy through replication fork collapse and mitotic catastrophe [47]. Importantly, our finding pertains to BRCA2 mRNA expression in tumors with wildtype BRCA2 alleles, not germline mutation carriers where haploinsufficiency drives this particular phenotype. Thus, elevated BRCA2 expression in our signature likely identifies highly proliferative tumors experiencing baseline replication stress, rendering them particularly susceptible to DNA-damaging chemotherapies.

The upregulation of E2F3 and MKI67 in pCR samples reflects the well-established proliferative paradox in chemotherapy response: highly proliferative tumors demonstrate increased sensitivity to cell cycle-targeting agents. This observation is consistent with the “chemosensitive proliferation” phenotype described in triple-negative breast cancers, where high proliferation rates correlate with favorable NAC response [48].

MED1’s role as a transcriptional co-activator and its enrichment in nuclear receptor binding functions implicate hormone signaling crosstalk in chemotherapy response. This finding is particularly relevant given that many breast cancers express hormone receptors [49], and interactions between endocrine signaling and chemotherapy efficacy remain incompletely understood.

The remaining genes, while exhibiting limited pathway enrichment, contributed significantly to predictive performance, suggesting involvement in biological processes not yet comprehensively annotated in current pathway databases. Genes such as ANKRD22, ANTKMT, ODAD4, and MISP3 represent understudied candidates for mechanistic investigation in chemotherapy response.

### 4.3. Molecular Subtype Distribution and Signature Independence

The distribution of molecular subtypes across response groups in both cohorts indicates that the 17-gene signature is not merely recapitulating known subtype biology but captures response-related biology that cuts across canonical classes. In the discovery cohort, among patients who achieved pCR, there were 38 TNBC, 29 HER2-enriched (HER2+ with or without hormone receptor expression), and 13 Luminal tumors reported, whereas among those with RD, there were 52 TNBC, 34 HER2-enriched, and 56 Luminal tumors. In the independent validation cohort, focusing on the two extreme response groups, the pCR group reported 14 TNBC, 18 HER2-enriched, and 5 Luminal tumors, while the RD group comprised 4 TNBC, 3 HER2-enriched, and 8 Luminal tumors.

These counts align with the established tendency of TNBC and HER2-positive tumors to achieve higher pCR rates than Luminal disease [50], yet the presence of Luminal tumors among pCR cases and TNBC tumors among RD cases in both cohorts indicates that the signature is not simply acting as a surrogate for molecular subtype, but is instead identifying particularly chemosensitive and chemoresistant biology within each subtype.

The overarching goal of this work was to develop a universal, pan-subtype predictor that can be applied in molecular heterogeneous clinical settings without imposing additional subtype-specific modeling layers, thereby prioritizing broad clinical applicability and reduced analytical complexity. At the same time, these findings highlight an important future direction: larger, subtype-balanced cohorts will be required to construct and rigorously evaluate subtype-stratified models, or to incorporate molecular subtype as an explicit covariate, to formally quantify the incremental value of subtype-aware frameworks beyond the universal 17-gene signature presented here.

### 4.4. Clinical Implications and Potential Applications

The 17-gene signature demonstrates clinically relevant predictive accuracy with high specificity (0.93 in discovery, 0.56 in validation at default threshold) and strong sensitivity for pCR detection (0.86 in validation). The high positive predictive value (0.95 in discovery) indicates particular reliability in identifying patients likely to achieve pCR, which could inform several clinical decisions. First, patients predicted to achieve pCR might be candidates for de-escalated surgery or omission of axillary lymph node dissection, reducing surgical morbidity while maintaining oncologic outcomes. Second, patients predicted to have residual disease could be considered for alternative neoadjuvant regimens, clinical trial enrollment, or upfront surgery followed by adjuvant therapy tailored to residual disease burden. Third, the signature could stratify patients in clinical trials by testing novel NAC combinations or de-escalation strategies.

The precision–recall AUC of 0.85 in external validation demonstrates strong performance in imbalanced classification scenarios representative of clinical practice, where pCR rates typically range from 20 to 40% depending on tumor subtype [51], indicating that predicted probabilities accurately reflect true pCR probabilities, enabling risk-stratified decision-making rather than binary classification.

Implementation of this signature in clinical practice would require the development of a standardized assay, validation in prospective cohorts, and integration into clinical decision support systems. The use of bulk RNA-seq, while comprehensive, presents challenges for rapid clinical turnaround. Future work should evaluate whether the 17-gene signature can be assessed using targeted platforms such as NanoString or qRT-PCR arrays, which offer faster, more cost-effective analysis suitable for routine clinical use.

### 4.5. Generalizability Beyond Breast Cancer

While developed specifically for breast cancer NAC response, the ensemble ML pipeline demonstrates potential for broader applicability to other solid tumors treated with neoadjuvant regimens. The hypothesis that this framework is universally applicable is supported by the identification of genes involved in fundamental cellular processes (DNA repair, cell cycle, and transcription) that mediate chemotherapy response across cancer types. Pathway enrichment in pancreatic cancer and bladder cancer pathways, alongside breast cancer-specific pathways, suggests shared molecular mechanisms of treatment response. However, cancer-specific validation would be essential, as tissue-specific biology, chemotherapy regimens, and tumor microenvironment characteristics may necessitate signature refinement. The framework’s strength lies not in direct transferability of the 17-gene signature, but in the reproducible methodology for ensemble feature selection and rigorous validation that can be applied to any cancer type with appropriate training data.

### 4.6. Integration with Mechanobiology Findings

Recent studies emphasize the importance of tumor mechanical forces in mediating chemotherapy resistance. Matrix stiffness and compressive forces can activate cell cycle and DNA repair pathways, influencing the genes, such as BRCA2 and E2F3, present in our signature [52]. Additionally, the mechanical regulation of the tumor–immune microenvironment may affect immune infiltration and response to therapy [53]. There is compelling experimental evidence that mechanical compression induces autophagy, fostering chemoresistance, which can be overcome through autophagy inhibition [54]. These insights highlight the link between transcriptomic predictors and physical TME features, supporting further research that integrates mechanical phenotyping with molecular analysis.

### 4.7. Limitations and Future Directions

Several limitations warrant acknowledgment. First, the retrospective design and reliance on publicly available datasets constrain sample sizes and limit control over data quality and clinical annotation. The validation cohort (*n* = 62) is modest, and while permutation testing confirmed statistical significance, larger independent validations are needed to refine performance estimates and assess performance across molecular subtypes. Second, both cohorts were derived from research datasets with potential selection biases not representative of general clinical populations. Prospective validation in consecutive patients undergoing NAC would provide more robust evidence for clinical utility.

Third, the study did not stratify by molecular subtype (ER+, HER2+, and triple-negative) or chemotherapy regimen, which likely influence treatment response mechanisms. Subtype-specific signatures may provide improved discrimination, though this requires substantially larger datasets. Fourth, the analysis did not incorporate clinical variables (tumor size, grade, and nodal status) that are established prognostic factors. Integration of genomic and clinical features in hybrid models may enhance predictive accuracy. Fifth, the lack of long-term survival data prevents assessment of whether the signature predicts disease-free or overall survival beyond pCR, which are the ultimate clinical endpoints.

Also, the decline in AUC from 0.97 in the discovery cohort to 0.78 in the external validation cohort reflects expected generalization loss and suggests model overfitting, despite the use of ensemble methods designed to reduce variance. The discovery phase yielded an EPV ratio of approximately 4.7 (80 pCR events across 17 genes), which falls below the traditional threshold of 10 often cited for logistic regression models. However, it is important to note that the EPV guidelines were developed primarily for continuous clinical risk prediction models and may not directly apply to classification tasks using ensemble ML methods, which employ inherent regularization through bootstrap aggregation, cross-validation, and feature subsampling. Nonetheless, the relatively low EPV increases the risk of model instability and variable selection artifacts. To mitigate these concerns, we implemented rigorous feature selection using stability-based recursive feature elimination with cross-validation, ensemble learning with multiple algorithms (RF, GB, SVM, KNN, and NN), and strict validation on an independent external cohort not used for any aspect of the model’s development.

Finally, our deliberate decision to focus validation on pCR versus extreme RD, rather than including all RD (i.e., minimal and moderate) categories deserves explicit acknowledgment. This design choice was motivated by clinical relevance: distinguishing patients who will achieve complete tumor eradication from those with substantial residual burden represents the most actionable contrast for treatment decision-making. However, excluding moderate responders simplifies the classification task and likely inflates performance metrics relative to a scenario requiring discrimination across the full spectrum of response. Future validation studies should assess model performance across all RD categories and in larger, more balanced cohorts to establish generalizability.

To that end, future research should focus on several priorities. First, prospective validation in large, multi-institutional cohorts with standardized protocols for sample collection, RNA extraction, and sequencing is essential for regulatory approval and clinical adoption. Second, development of a cost-effective, rapid-turnaround assay platform (e.g., NanoString or qRT-PCR) would facilitate clinical implementation. Third, mechanistic studies investigating the functional roles of understudied genes (ANKRD22, ANTKMT, ODAD4, and MISP3) could reveal novel therapeutic targets. Fourth, integration with other data modalities (proteomics, radiomics, and circulating tumor DNA) in multimodal prediction models may further improve accuracy. Finally, application of this ensemble methodology to other cancer types would test the hypothesis of universal applicability.

### 4.8. Methodological Considerations

The ensemble ML framework employed here offers several advantages over traditional single-algorithm approaches. By integrating predictions from multiple algorithms with distinct inductive biases (i.e., tree-based methods (RF and GB), kernel methods (SVM), instance-based learning (k-NN), and DL (NN)), the ensemble captures diverse aspects of the data structure and reduces vulnerability to algorithm-specific overfitting. The stacked ensemble meta-learning approach, which trains a higher-level classifier on base classifier outputs, optimizes combination weights rather than using simple averaging, further enhancing performance.

The stringent consensus feature selection, requiring genes to appear in all five algorithms across three independent data configurations, prioritizes stability over marginal performance gains. This conservative approach addresses a fundamental challenge in biomarker discovery: features selected by aggressive variable selection methods often fail to replicate in independent cohorts, with studies showing that 93% of features identified in single datasets fail validation [40]. While this strategy reduces the signature to genes from an initial 250, the resulting features demonstrate extraordinary robustness, as evidenced by successful external validation.

The use of ComBat batch correction and probability calibration in the validation phase addresses critical challenges in external validation. Batch effects between discovery and validation cohorts can artificially inflate or deflate performance estimates; ComBat harmonization ensures that classifier performance reflects the biological signal rather than technical artifacts. Isotonic regression calibration adjusts for distributional shifts between training and validation data, ensuring that predicted probabilities remain interpretable and actionable.

## 5. Conclusions

This study presents a rigorously validated 17-gene signature for predicting NAC response in breast cancer, developed through ensemble ML integrating five independent classification algorithms. The signature achieved strong discrimination in the discovery cohort (AUC = 0.97) and demonstrated robust external validation (AUC = 0.78), with particularly high sensitivity for pCR detection. Pathway enrichment analysis revealed biologically coherent mechanisms involving DNA repair (BRCA2), cell cycle regulation (E2F3, MKI67), transcriptional control (MED1), and cytoskeletal dynamics (FARP2), providing mechanistic insights into chemotherapy response.

The ensemble feature selection methodology, requiring genes to appear in all five classifiers across multiple data configurations, ensures exceptional stability and addresses reproducibility challenges in biomarker discovery. External validation in an independent cohort with different sequencing protocols demonstrates generalizability beyond the discovery dataset, a critical requirement for clinical translation. The use of publicly available data and open-source algorithms enhances reproducibility and facilitates rapid clinical adoption.

This 17-gene signature provides a biologically grounded, clinically relevant tool for NAC response prediction with potential applications in treatment de-escalation strategies, clinical trial stratification, and alternative therapy selection for patients unlikely to achieve pCR. The ensemble ML framework demonstrates broader applicability to other solid tumors treated with NAC, supporting the hypothesis of universal applicability across cancer types. Prospective validations in large, multi-institutional cohorts, and the development of rapid-turnaround clinical assays represent essential next steps for clinical implementation.

## Figures and Tables

**Table 2:** Functional enrichment summary of representative genes across annotation databases.

Gene	Enriched Pathways	KEGG	GO BP	Reactome
E2F3	8	Yes	Yes	Yes
BRCA2	7	Yes	Yes	Yes
MED1	5	No	Yes	No
PITPNB	2	No	No	No
H1-1	2	No	No	Yes
FARP2	1	No	No	Yes

GO BP = Gene Ontology Biological Process.

**Table 3:** Top enriched biological processes and pathways among signature genes.

Category	Gene(s)	Term/Pathway	*p*-Value	Combined Score
GO:BP	MED1, BRCA2	Negative regulation of epithelial cell proliferation	0.00159	251.70
GO:BP	MED1, E2F3	Transcription initiation at RNA polymerase II promoter	0.00187	225.39
GO:BP	BRCA2	Protein localization to telomere	0.00424	1705.22
GO:MF	MED1	LBD domain binding	0.00424	1705.22
KEGG	E2F3, BRCA2	Pancreatic cancer	0.00187	225.39
KEGG	E2F3, BRCA2	Breast cancer	0.00679	89.50
Reactome	E2F3	G2 phase	0.00424	1705.22

GO:BP = Gene Ontology Biological Process; GO:MF = Gene Ontology Molecular Function.

## Data Availability

The raw data supporting the conclusions of this article are publicly available. The discovery cohort (GSE163882) can be accessed at https://www.ncbi.nlm.nih.gov/geo/query/acc.cgi?acc=GSE163882 (accessed on 2 October 2025) and the validation cohort (GSE240671) can be accessed at https://www.ncbi.nlm.nih.gov/geo/query/acc.cgi?acc=GSE240671 (accessed on 2 October 2025). All analysis code, including data preprocessing pipelines, differential expression analysis scripts, ensemble machine learning implementation, and external validation procedures, are openly available at https://github.com/stelioslamprou37/cancer-nac-ensemble (accessed on 20 November 2025) under an MIT license. The repository includes the following: 1. R scripts for TMM normalization and DESeq2 differential expression analysis; 2. R code for ensemble feature selection across five classifiers; 3. Trained stacked ensemble model weights and configuration files; and 4. Complete list of 250 differentially expressed genes and 17-gene consensus signature.

## Abbreviations

The following abbreviations are used in this manuscript:

NAC	Neoadjuvant Chemotherapy
pCR	Pathological Complete Response
RD	Residual Disease
TMM	Trimmed Mean of M-values
FDR	False Discovery Rate
log2FC	Log2 Fold Change
RF	Random Forest
GB	Gradient Boosting
SVM	Support Vector Machine
k-NN	k-Nearest Neighbors
NN	Neural Network
AUC	Area Under the Curve
ROC	Receiver Operating Characteristic
PR	Precision–Recall
ECM	Extracellular Matrix
CPM	Counts Per Million
DESeq2	Differential Expression analysis using Shrinkage Estimation for Dispersed Count Data
EPV	Events-Per-Variable
PPV	Positive Predictive Value
NPV	Negative Predictive Value
OS	Overall Survival
DFS	Disease-Free Survival
ML	Machine Learning
DL	Deep Learning
ER+	Estrogen Receptor-Positive
HER2+	Human Epidermal Growth Factor Receptor 2-Positive
GO	Gene Ontology
KEGG	Kyoto Encyclopedia of Genes and Genomes
BP	Biological Process
MF	Molecular Function
EMT	Epithelial-to-Mesenchymal Transition
CSC	Cancer Stem Cell
TME	Tumor Microenvironment
CAF	Cancer-Associated Fibroblast
LASSO	Least Absolute Shrinkage and Selection Operator
